# Assessment of Areca Nut Bioactivities in Western Diet-Induced Mice NAFLD Model

**DOI:** 10.3390/nu15102403

**Published:** 2023-05-21

**Authors:** Shuhan Yi, Keyu Chen, Kozue Sakao, Makoto Ikenaga, Yuanliang Wang, De-Xing Hou

**Affiliations:** 1 The United Graduate School of Agricultural Sciences, Kagoshima University, Kagoshima 890-0065, Japan; yishuhan.changsha@foxmail.com (S.Y.); sakaok24@agri.kagoshima-u.ac.jp (K.S.); ikenaga@agri.kagoshima-u.ac.jp (M.I.); 2 School of Physical Education and Health, Health Service and Management, Hunan University of Technology and Business, Changsha 410205, China; 2907@hutb.edu.cn; 3 Faculty of Agriculture, Kagoshima University, Kagoshima 890-0065, Japan; 4 College of Food Science and Technology, Hunan Agricultural University, Changsha 410128, China; wangyuanliang@hunau.edu.cn

**Keywords:** areca nut polyphenol, arecoline, obesity, HMGCR, gut microbiota

## Abstract

The areca nut is often consumed as a chewing food in the Asian region. Our previous study revealed that the areca nut is rich in polyphenols with high antioxidant activity. In this study, we further assessed the effects and molecular mechanisms of the areca nut and its major ingredients on a Western diet-induced mice dyslipidemia model. Male C57BL/6N mice were divided into five groups and fed with a normal diet (ND), Western diet (WD), WD with areca nut extracts (ANE), areca nut polyphenols (ANP), and arecoline (ARE) for 12 weeks. The results revealed that ANP significantly reduced WD-induced body weight, liver weight, epididymal fat, and liver total lipid. Serum biomarkers showed that ANP ameliorated WD-enhanced total cholesterol and non-high-density lipoprotein (non-HDL). Moreover, analysis of cellular signaling pathways revealed that sterol regulatory element-binding protein 2 (SREBP2) and enzyme 3-hydroxy-3-methylglutaryld coenzyme A reductase (HMGCR) were significantly downregulated by ANP. The results of gut microbiota analysis revealed that ANP increased the abundance of beneficial bacterium *Akkermansias* and decreased the abundance of the pathogenic bacterium *Ruminococcus* while ARE shown the opposite result to ANP. In summary, our data indicated that areca nut polyphenol ameliorated WD-induced dyslipidemia by increasing the abundance of beneficial bacteria in the gut microbiota and reducing the expressions of SREBP2 and HMGCR while areca nut ARE inhibited this improvement potential.

## 1. Introduction

The areca nut (*Areca catechu*) has been a chewing food in Asian areas for a long time [1]. However, the areca nut is classified as a class I carcinogen by the IARC since frequent consumption of the areca nut is positively associated with the development of oral diseases, and the areca nut alkaloids were reported to play a crucial role in causing oral diseases [2]. ARE is one of the main alkaloids in the areca nut and has been reported to inhibit adipogenic differentiation of preadipocytes and induce lipolysis in adipocytes. Animal data showed that arecoline reduced mice’s body weight at low doses without adverse effects [3,4,5]. On the other hand, the ARE base has been reported as a carcinogen in the development of cancer, and the safety of ARE remains controversial [6]. Another major component of the areca nut is polyphenol, with stronger antioxidant properties [7,8]. The results from our previous study combined with those of other groups revealed that areca nut polyphenol had many bioactivities such as antioxidant, anti-inflammatory, and anti-atherosclerotic effects [9].

Obesity is a global health problem. A major cause of obesity is an unhealthy diet, often high in fat and sugar. In 2016 alone, 39% of adults were overweight and 13% were obese. Being overweight and obese are major risk factors for many chronic diseases [10]. For example, being overweight is often associated with non-alcoholic fatty liver disease (NAFLD) and leads to diabetes. NAFLD is strongly associated with metabolic syndrome, which can lead to non-alcoholic steatohepatitis, cirrhosis, and liver cancer as the disease progresses [11]. The pathogenesis of NAFLD is related to many factors, such as dietary habits, obesity, gut microbiota changes, body lipid homeostasis disturbances, and excessive accumulation of triglycerides (TG) and other lipids [12]. NAFLD is characterized by the accumulation of TG and free cholesterol (FC) [13]. Animal studies have shown that experimental induction of FC accumulation can promote steatohepatitis and liver fibrosis [14,15]. Cholesterol synthesis occurs in the endoplasmic reticulum and is tightly regulated by the enzyme 3-hydroxy-3-methylglutaryl coenzyme A reductase (HMGCR), which is a class of rate-limiting enzyme in the process of cholesterol synthesis [16,17]. HMGCR activity was positively correlated with hepatic free cholesterol accumulation and the severity of NAFLD [18]. The SREBP2 is a key regulator of cholesterol homeostasis, and intracellular cholesterol synthesis is controlled by a complex feedback mechanism dependent on the transcription factor SREBP-2 [19]. When experimental induction leads to metabolic dysregulation, upregulation of SREBP2 is positively correlated with the severity of NAFLD [20]. The expressions of HMGCR and SREBP2 were elevated in NAFLD compared to normal people [21,22]. The AMP-activated protein kinase (AMPK) is also associated with many metabolic diseases. In NAFLD, the phosphorylation of AMPK is inhibited [23,24], which can directly inhibit the expression levels of HMGCR and SREBP2 in high-fat-diet-induced obesity [25,26].

Gut microbiota is closely related to health, and many diseases are associated with changes in gut microbiota [27]. Unhealthy dietary habits can affect the gut microbiota, for example, long-term high-fat and high-sugar diets can lead to the dysbiosis of gut microbiota, and further promote the development of NAFLD [28]. Gut microbiotas from mice with hyperglycemia and high levels of pro-inflammatory cytokines were transferred to germ-free mice and fed the same high-fat diets. The results showed that gut microbiotas promoted the development of NAFLD in recipient mice, but it did not occur in the gut microbiotas from healthy mice [29].

According to the above information and current research, the areca nut and its polyphenols have the ability to prevent unhealthy diet-induced disorders, which may be regulated by gut flora. To determine which of the areca nut’s components contributes to areca nut biofunctions, we created whole ANE, ANP, and ARE as experiment materials. The WD-induced mouse NAFLD model was utilized to study changes in lipid metabolism as well as alterations in the gut microbiota following supplementation with these three extracts. The present study will give a reliable understanding of the function and safety of the whole areca nut and its individual constituents.

## 2. Materials and Methods

### 2.1. Preparation of Whole Areca Nut Powder and Its Polyphenols as Well as ARE

The raw areca nut was obtained in January 2018 in Wanning City, Hainan Province, China. Areca nut powder and ANP were prepared as described previously [30,31]. In brief, the fresh areca nuts were freeze dried, then pulverized into powder. To get crude polyphenols, the areca nut powder was extracted with 50% (*v*/*v*) ethanol at 68 °C for 48 min and freeze dried as ANE. The major polyphenols in ANE were identified as catechins (2060.44 ± 18.24 µg/mL) and proanthocyanidin B1 (2510.18 ± 62.40 µg/mL) [30,31]. The crude polyphenols were further purified with XAD-7 macroporous resin by washing with 50% ethanol and freeze dried as ANP. The total polyphenol contents in ANP were estimated as 80% by the Folin–Ciocalteu method. AREs with 98% purity were purchased from Shanghai Yuanye Bio-Technology Co., Ltd. (Shanghai, China).

### 2.2. Animal Experiment Design

**Figure 1:**
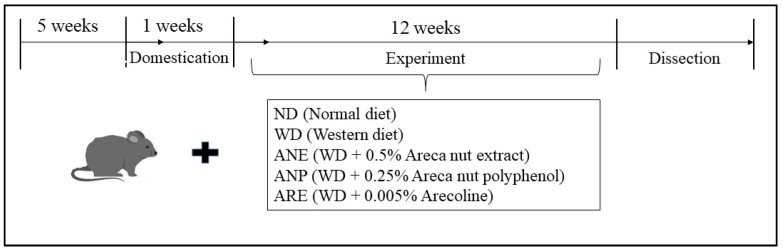
Design of animal experiments.

Animal experiments were performed in compliance with the Guidelines of the Animal Care and Use Committee of Kagoshima University and approved by the Animal Ethics Committee of Kagoshima University (Permission No. A12005). Male C57BL/6N mice aged 5 weeks were obtained from Japan SLC Inc. (Shizuoka, Japan). The mice were raised individually in cages with free access to water and food with controlled light (12 h) and temperature (24 °C). Every experiment was planned to use the lowest mouse number possible, minimizing experiment pain according to the Animal Ethics Committee. The mice were randomly assigned to 5 groups (4/each group) which were fed with ND, WD, WD + 0.5% ANE, WD + 0.25% ANP, or a WD + 0.005% ARE after acclimation for a week. The 0.5% experimental dose of ANE was set according to the results of previous experiments with safe doses [31]. The contents of polyphenols and ARE in the ANE were measured respectively at 50% and 1%. To clarify the effects of polyphenols and alkaloids which are equal to content in a 0.5% dose of areca nut (ANE), we also designed mouse groups supplemented with 0.25% ANP or 0.005% ARE (Figure 1). The nutrient composition of the diets is shown in Appendix A Table A1. ND and WD designs were based on our previous studies [32]. The mice fed with ND were supplied with normal drinking water while mice fed WD, ANE, ANP, or ARE were supplied with 4% sugar water containing 18.9 g/L sucrose and 23.1 g/L fructose.

### 2.3. Measurement of Serum Biochemical Indicators

Isoflurane was used to stupefy the mice for reducing pain. Mouse blood was collected in the tube with a coagulant (Separable microtubes, Fuchigami 170720, Japan), and then coagulated for 30 min at 24 °C. The serum samples were separated by centrifugation at 1000× *g* for 10 min and stored at −80 °C until use. An automated analyzer for Clinical chemistry (Spotchem EZ SP-4430, Arkray, Kyoto, Japan) was used to assess the levels of glutamic-oxalacetic transaminase (GOT), glutamic-pyruvic transaminase (GPT), triacylglycerol (TG), total cholesterol (T-Cho), and HDL cholesterol. The non-HDL was computed by the formula “T-Cho—HDL” [33].

### 2.4. Analysis of Liver Lipid Rate and Histomorphology

Mice livers were fixed in 4% paraformaldehyde for 24 h and dehydrated with 30% sucrose water. Livers were embedded in O.C.T compound (Sakura Finetek, Tokyo, Japan) and sectioned into frozen sections (Yamato, Saitama, Japan). Liver sections were stained with hematoxylin-eosin and viewed under a microscope (Keyence, Tokyo, Japan). A modified Soxhlet method was used to assess the liver lipid content. Liver tissue was mixed with n-hexane and homogenized until crushed. After centrifuging at 1000× *g* for 10 min to collect the supernatant, n-hexane was then removed from the supernatant, and the liver lipid was weighed. The liver lipid percentage formula was used to estimate the ratio of liver lipid weight and liver weight.

### 2.5. Western Blotting Analysis

The antibodies were anti-phosphorylated-AMPKα (p-AMPKα), anti-AMPKα, anti-SREPB2, anti-HMGCR, anti-β-actin, and anti-rabbit and were purchased from Cell Signaling Technology (Boston, MA, USA). Liver tissue preparation and Western blot analysis were performed according to our previous method [32]. Briefly, liver tissue was homogenized in a homogenizer (Speedmill plus, Analytik Jena, Germany) with modified RIPA buffer (50 mM Tris-HCl (pH8.0), 150 mM NaCl, 1 mM EDTA, 1% Nonidet P-40, 0.25% Na-deoxycholate, 1 mM sodium fluoride, 1 mM sodium orthovanadate, 1 mM phenylmethylsulphonyl fluoride), plus a proteinase inhibitor cocktail (Nacalai Tesque, Inc., Kyoto, Japan). The supernatants were collected after centrifuging for 15 min at 14,000 rpm and 4 °C. The protein content of the lysates was measured with a protein assay kit (Bio-Rad Hercules, CA, USA) as instructed. Proteins were separated onto 10% sodium dodecyl sulfate polyacrylamide gels and then transferred to polyvinylidene difluoride membranes and blocked with 5% skim milk powder in phosphate-buffered saline with Tween-20. The mentioned primary monoclonal antibody was incubated overnight at 4 °C, followed by a secondary antibody diluted 2000 times in skimmed milk for an hour at room temperature. Blots were visualized using a Bio-Rad LumiVision PRO system with an enhanced chemiluminescence kit (Taitec, Saitama, Japan).

### 2.6. Gut Microbiota Analysis

Fecal DNA extract and 16S rRNA gene sequencing were done as described in our previous paper [34]. In brief, all mouse feces from week 18 were immediately collected and preserved at −80 °C. Fecal genomic DNA was isolated using the Fast DNA spin kit (MP Biomedicals). DNA concentration was measured with a Nanodrop 2000c spectrophotometer (Thermo Fisher Scientific Inc., Waltham, MA, USA). The 16S rRNA genes were amplified using 16S rRNA gene V3 region primers (Illumina, San Diego, CA, USA). The list of filtered sequences by Qiime2 (ver. 2020.2) and Greengene (ver. 13_8), which contained sequences with 97% identity, were used to select operational taxonomic units.

### 2.7. Statistical Analysis

The data were expressed as mean ± SD and analyzed using one-way ANOVA (SPSS, 2010), and the differences among treatment groups were evaluated using least significant difference and Duncan’s multiple range tests, with a significance threshold of *p* < 0.05 or *p* < 0.01 [35].

## 3. Results

### 3.1. Effects of the Areca Nut and Its Ingredients on WD-Induced Body Weight and Liver Weight

**Figure 2:**
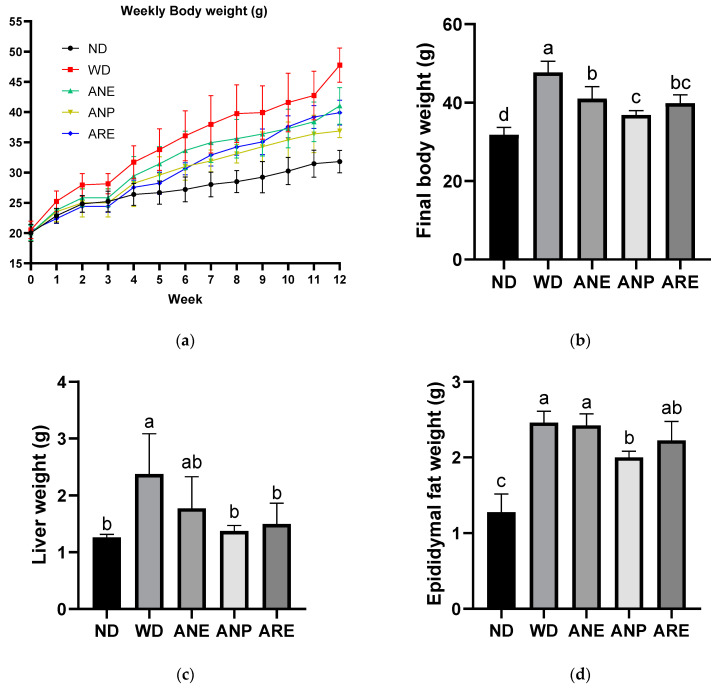
Effect of the areca nut and its ingredients on WD-induced body weight and liver weight on the bodies of mice. The data are shown as means ± SD. (**a**) Weekly body weight. (**b**) Final body weight. (**c**) Liver weight. (**d**) Epididymal fat weight. The graph’s various letters denoted significance (*p* < 0.05).

To explore the effect of areca nuts on the prevention of WD-induced obesity and dyslipidemia, mice were fed with three different samples of ANE, ANP, and ARE for 12 weeks. As shown in Figure 2a,b, the final body weights of all areca nut groups were significantly decreased compared to the WD group. ANP most significantly decreased the body weight in the three sample groups in Figure 2b. Moreover, WD-induced liver weight and epididymal fat were significantly decreased by ANP although ARE also significantly decreased liver weight (Figure 2c,d). These data demonstrated that the areca nut could ameliorate WD-induced overweight, and ANP acted a master ingredient for this effect.

### 3.2. Effects of the Areca Nut and Its Ingredients on WD-Induced Fatty Liver

**Figure 3:**
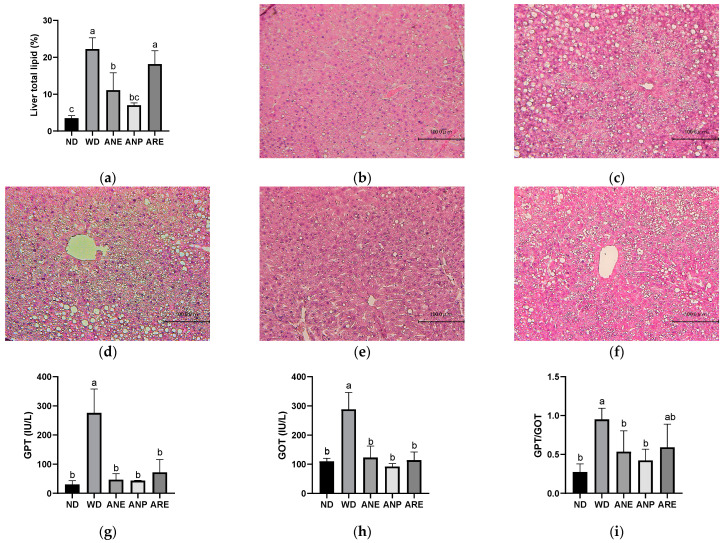
Effects of the areca nut and its ingredients on WD-induced fatty liver. Effect of the areca nut on the hepatic H&E staining and hepatic indices in serum of mice. (**a**) Liver total lipid rate. The data are shown as means ± SD. The hepatic H&E staining in ND (**b**), WD (**c**), ANE (**d**), ANP (**e**), ARE (**f**). The serum levels of GPT (**g**), GOT (**h**), and the ratio of GPT and GOT (**i**). The graph’s various letters denoted significance (*p* < 0.05).

To investigate the effect of the areca nut and its ingredients on WD-induced fatty liver, we measured liver total lipid rate and liver injury biomarkers. As shown in Figure 3a, the liver’s total lipid rate was increased by WD, and significantly reduced by ANE and ANP, but not ARE.

Furthermore, the hepatic H&E staining results indicated that the lipid droplets were markedly increased in WD and markedly reduced in ANP, although ANE and ARE showed a reduced trend but not as clear as ANP (Figure 3b,f). To confirm this, we further measured the serum level of GPT (liver specific) and GOT (most organs), which are two important indicators of liver injury and injury to other organs. As shown in Figure 3g,h, GPT in WD was 5-fold higher than in ND and GOT in WD was almost 3-fold higher than it in ND. ANP, ARE, and ANE significantly decreased the serum levels of GPT and GOT. However, the GPT/GOT ratio, an index of fatty liver injury, was only significantly reduced by ANP and ANE, but not ARE. These data indicate that fatty liver induced by WD was improved by ANP, not by ARE (Figure 3i). ANP of the areca nut reduced the accumulation of lipid droplets in the liver and improved liver injury.

### 3.3. Effects of the Areca Nut and Its Ingredients on WD-Induced Dyslipidemia

**Figure 4:**
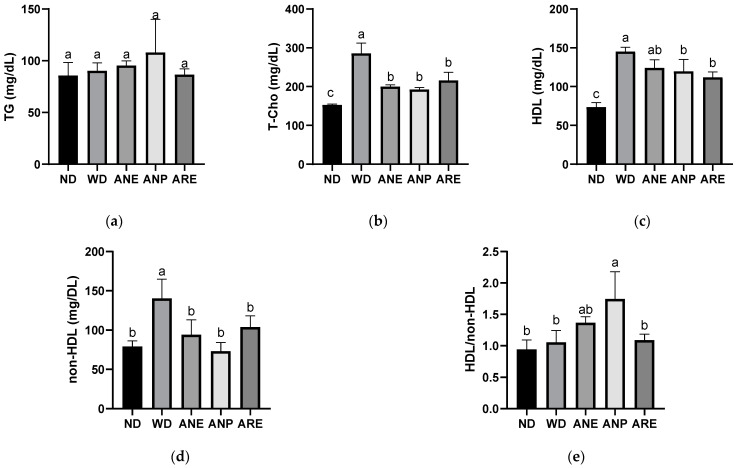
Effects of the areca nut and its ingredients on WD-induced dyslipidemia. (**a**) Serum TG. (**b**) Serum T-Cho. (**c**) Serum HDL. (**d**) Serum non-HDL. (**e**) The ratio of HDL and non-HDL. The data are shown as means ± SD. The graph’s various letters denoted significance (*p* < 0.05).

To investigate the effect of the areca nut on WD-induced dyslipidemia, we measured the levels of lipid metabolism indices. Although there was no significant difference in the serum level of TG (Figure 4a), the T-Cho (Figure 4b), HDL (Figure 4c), and non-HDL (Figure 4d) were significantly increased by WD, and significantly reduced by ANP and ARE. ANE only reduced significantly T-Cho and non-HDL. The ratio of HDL/non-HDL (Figure 4e) was only increased by ANP. These results suggested that ANP acted as the bioactive ingredient to improve WD-increased dyslipidemia.

### 3.4. Effects of the Areca Nut and Its Ingredients on WD-Induced Cholesterol Synthesis

**Figure 5:**
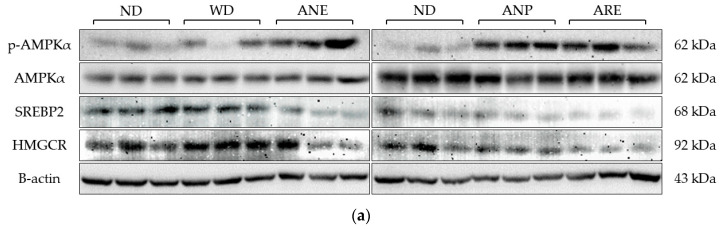

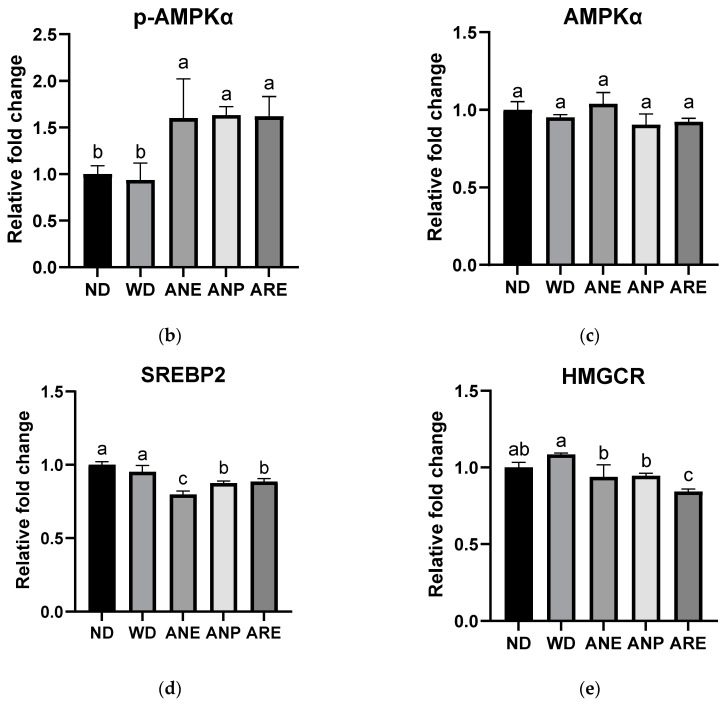
Effects of the areca nut and its ingredients on WD-induced cholesterol synthesis. (**a**) The western blot results of mice liver protein. (**b**) The relative fold change of p-AMPKα. (**c**) The relative fold change of AMPKα. (**d**) The relative fold change of SREBP2. (**e**) The relative fold change of HMGCR. The data are shown as means ± SD. The graph’s various letters denoted significance (*p* < 0.05).

According to the serum results, the ANP reduced WD-induced T-Cho levels. Therefore, we detected and quantified the key factors involved in cholesterol synthesis by Western blotting. Due to large number of samples, we performed Western blotting in two plates using the same ND as the control (Figure 5a). The blot density was then quantified and normalized with ND. As shown in Figure 5b, WD did not activate p-AMPKα while ANE, ANP and ARE activated p-AMPKα (Figure 5b). The total AMPKα had no significant change in all groups (Figure 5c). The expression levels of SREBP2 and HMGCR were significantly decreased by the ANE, ANP, and ARE (Figure 5d,e). These results revealed that the areca nut and its ingredients might inhibit WD-induced cholesterol synthesis.

### 3.5. Effect of the Areca Nut and Its Ingredients on WD-Induced Gut Microbiota in Mice

**Figure 6:**
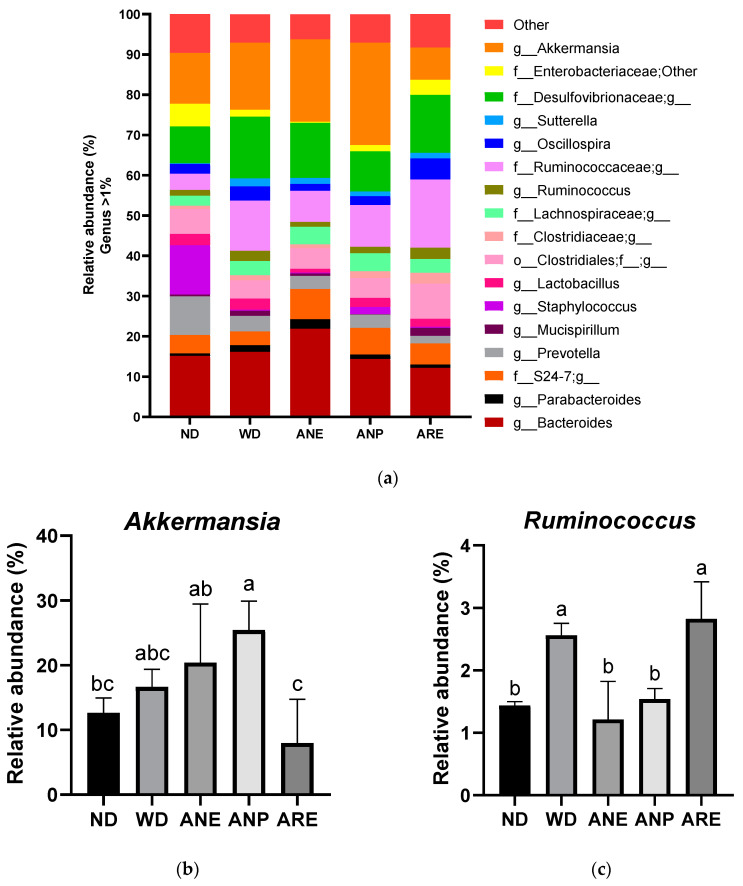
Effect of the areca nut and its ingredients on WD-induced gut microbiota in mice. The data are shown as means ± SD. (**a**) The genus level results of gut microbiota. (**b**)The abundance of *Akkermansia*. (**c**) The abundance of *Ruminococcus*. The graph’s various letters denoted significance (*p* < 0.05).

To assess the effect of the areca nut and its ingredients on gut microbiota, fecal samples were collected at the end of the experiment (18 weeks of age). The relative abundances of bacteria in feces identified by 16S rRNA sequencing are shown in Figure 6a. The areca nut and its ingredients showed different effects on mice gut microbiota. In particular, the abundance of *Akkermansia* was significantly increased by ANP and decreased by ARE, compared to ND (Figure 6b). On other hand, a significant decrease in the abundance of *Ruminococcus* was observed in ANE and ANP, but was not changed in ARE compared with WD (Figure 6c).

**Figure 7:**
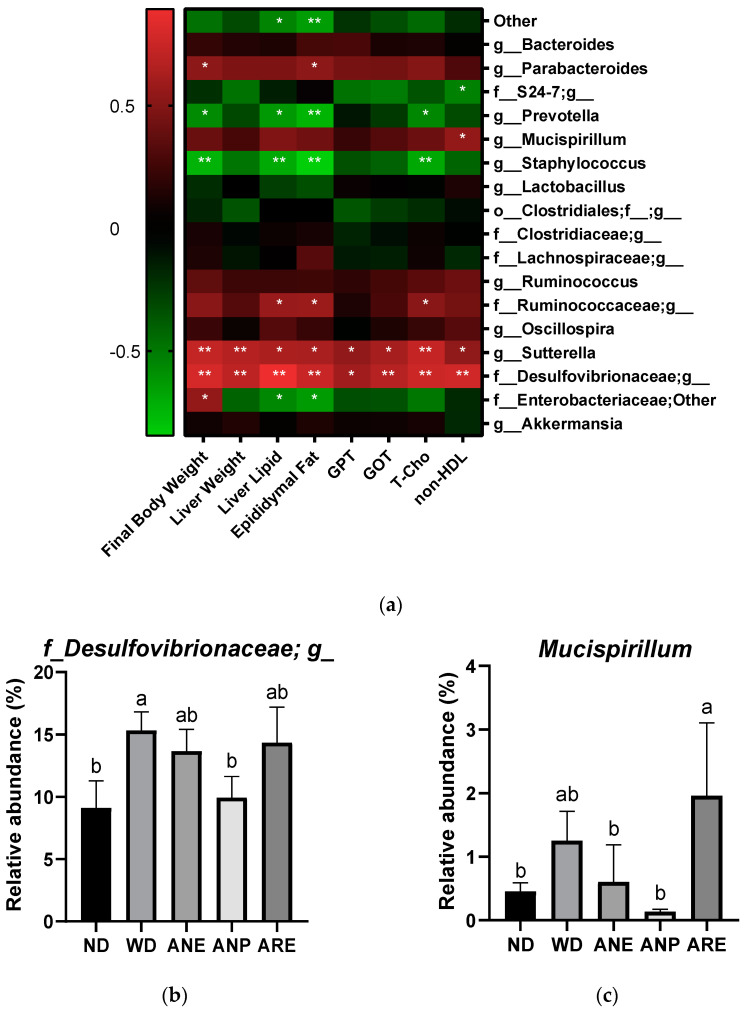
The Pearson correlation analysis of the areca nut on gut microbiota in mice. The data are shown as means ± SD. (**a**) The heat map of the Pearson correlation analysis of physiological indicators and genus-level gut microbiota in mice. (**b**) The abundance of *f_Desulfovibrionaceae;g*. (**c**) The abundance of *Mucispirillum*. The graph’s various letters denoted significance (*p* < 0.05). * indicate significance (*p* < 0.05), ** indicate extremely significance (*p* < 0.01).

Finally, we used Pearson correlation analysis to explore the relationship between the gut microbiome community and serum biochemical profiles. As shown in Figure 7a, significant negative correlations were observed in final body weight, liver lipid, epididymal fat T-Cho, and the relative abundance of *g_Prevotella* and *g_Staphylococcus*. On the other hand, significantly positive correlations were observed in final body weight, liver weight and fat, epididymal fat, GPT, GOT, T-Cho, and non-HDL, and the relative abundance of *g_Sutterella*, and *f_Desulfovibrionaceae;g*. ANP decreased the abundances of these two bacteria with significance. It is noteworthy that *f_Desulfovibrionaceae;g* was correlated with NAFLD [36].

Taken together, ANP had a promotive effect on probiotics such as *Akkermansia* and *Ruminococcus*, and an inhibitory effect on pathogenic bacteria such as *g_Sutterella*, and *f_Desulfovibrionaceae;g_.* On the other hand, ARE had the opposite effect to ANP on these bacteria.

## 4. Discussion

The areca nut is a highly consumed tropical fruit in China, India, and Southeast Asian countries. However, the areca nut contains both bioactive polyphenols and ARE. ARE has been also reported to cause the accumulation of reactive oxygen species (ROS) which further cause cholesterol accumulation in the liver, leading to NAFLD [37,38]. Previous study revealed that ANE containing both polyphenols and ARE had a ROS scavenging ability and antioxidant ability [39,40], and ANP containing only polyphenols significantly scavenged LPS-induced ROS [41]. To provide a reliable understanding of the function and safety of the areca nut, we assessed the effects of whole areca nut extract, polyphenol extract, and ARE on a WD-induced NAFLD mouse model by investigating the changes in metabolic characters and gut microbiota.

In morphological traits, APN revealed a significant ameliorative effect in most morphological traits including WD-induced high final body weight, liver weight, and epididymal fat. ARE only significantly ameliorated WD-induced high final body weight and liver weight. The body weights of mice were reduced by ARE, which is consistent with previous studies [3,4,5]. ANE only significantly ameliorated WD-induced high final body weight (Figure 2), which is consistent with previous studies [31]. These data demonstrated that the areca nut could ameliorate WD-induced excess weight, and its polyphenol acted a master ingredient for this effect. Interestingly, a study reported an association between areca nut chewing and obesity that was due to increased appetite, rather than the fact that the areca nut itself causes obesity [42].

Regarding WD-induced fatty liver, ANP revealed a significant ameliorative effect in liver lipid rate and accumulation of lipid droplets in the liver, which were related to being overweight and NAFLD [43]. ANE only significantly ameliorated a WD-induced high liver lipid rate. However, ARE did not for both. This result implies that the lipid reduction effect of the areca nut is the most significant for ANP. Moreover, GOT, and GPT, two important indicators of liver damage, were detected [44]. The GPT/GOT ratio, an index of fatty liver injury, was only significantly reduced by ANP and ANE, but not ARE. This means that ARE does not improve liver injury caused by WD. These data indicated that fatty liver induced by WD was improved by ANP, not by ARE (Figure 3).

Next, we investigated the effect of the areca nut on WD-induced dyslipidemia by measuring the levels of lipid metabolism indices. Some previous studies have revealed that T- Cho and non-HDL are strongly associated with diseases such as cardiovascular disease, NAFLD, and so on [45,46]. In contrast, HDL is a good class of cholesterol that is negatively associated with the development of diseases such as being overweight and NAFLD [46]. The ratio of HDL/non-HDL (Figure 4e) was only increased by ANP, which means that ANP mainly reduced non-HDL and has a less reducing effect on HDL, although the serum levels of WD-induced high T-Cho (Figure 4b) and non-HDL (Figure 4d) were significantly reduced by all of ANE, ANP, and ARE. These results suggested that ANP acted as the bioactive ingredient to improve WD-increased dyslipidemia. AMPKα-SREBP2-HMGCR are reported to play an important role in obesity-related diseases [47,48]. Our cellular signaling analysis revealed that the areca nut and its ingredients inhibited WD-induced cholesterol synthesis by increasing p-AMPKα expression to inhibit SREBP2 and the HMGCR pathway at least. These data provided partial evidence for the molecular mechanisms of anti-obesity effects of areca nuts.

Finally, the areca nut and its ingredients showed different effects on mouse gut microbiota. In particular, the abundance of *Akkermansia* was increased significantly by ANP and decreased by ARE, compared to ND (Figure 6b). These data indicated that the areca nut improved the abundance of *Akkermansia* due to ANP. *Akkermansia* is a probiotic associated with lipid metabolism and is increased by many polyphenols [49,50]. Reduced abundance of *Akkermansia* bacteria has been associated with a variety of diseases in both mouse models and population studies [51]. On other hand, a significant decrease in the abundance of *Ruminococcus* was observed in ANE and ANP, but not in ARE compared with WD (Figure 6c). *Ruminococcus* is a genus of bacteria associated with obesity and cardiovascular disease [52,53]. Furthermore, Pearson correlation analysis revealed significantly positive correlations in high final body weight, liver weight and fat, epididymal fat, T-Cho, non-HDL, GPT and GOT, and the relative abundance of *f_Desulfovibrionaceae; g*. Interestingly, ANP significantly reduced its abundance; ANE tended to reduce its abundance without significance. However, ARE significantly enhanced its abundance. It is noteworthy that *f_Desulfovibrionaceae* and *Mucispirillum* were correlated with NAFLD [36]. Taken together, ANP acted as prebiotics for gut microbiota and reduced the abundance of pathogenic bacteria. ARE was harmful for gut microbiota.

ARE is the primary active ingredient responsible for the central nervous system effects of the areca nut. Thus, we carefully observed the behaviors of all mice during the experimental period. No abnormality statement and no mortality were observed in all mice fed with ANP, ANE, and ARE, compared with ND and WD. Thus, we considered that the feeding dose in this study did not cause any abnormality of behavior of the mice.

Taken together, our data showed that areca nut crude containing both polyphenol and ARE had a potential preventive effect on obesity, liver injury, as well as gut microbiota. However, the effects of areca nut crude were not as significant as areca nut polyphenol, and ARE had no such effects. These data suggested that ARE in areca nut crude might partially inhibit the improvement potential of areca nut polyphenol on WD-induced obesity, liver injury, and gut microbiota. On the other hand, the ethanol extract of the areca nut used in this study did not involve areca nut crude fiber and other nutritional components. It is still hard to completely simulate the effect of the whole areca nut on human health when people chew the whole areca nut.

## 5. Conclusions

In conclusion, our works show that ANE and ANP ameliorated WD-induced obesity via activating p-AMPKα to inhibit the SREBP2 and HMGCR expressions and by improving the gut microbiota. On the other hand, ARE had an adverse effect on the gut microbiota. These data provide evidence for understanding the polyphenol benefit function and ARE side effect in the areca nut.

## Data Availability

No data available.

## Appendix A

**Table A1:** Dietary compositions of each group.

Components (%)	ND	WD			
	-	-	ANE	ANP	ARE
Lard	3	30	30	30	30
Soybean oil	3	3	3	3	3
Corn starch	43	15	15	15	15
Casein	21	21	21	21	21
Sucrose	20	20	20	20	20
Cellulose	5	5	5	5	5
Mineral Mix	3.5	3.5	3.5	3.5	3.5
Vitamin Mix	1	1	1	1	1
Cholesterol	0	1	1	1	1
Choline Bitartrate	0.2	0.2	0.2	0.2	0.2
Methionine	0.3	0.3	0.3	0.3	0.3
Areca nut extract (ANE)	0	0	0.5	0	0
Areca nut polyphenol (ANP)	0	0	0	0.25	0
Arecoline (ARE)	0	0	0	0	0.005
Total calories (kcal/100 g)	371	520	520	520	520

ND: normal diet, WD: western diet, ANE: western diet + areca nut extract, ANP: western diet + areca nut polyphenol, ARE: western diet + arecoline.
